# Mebendazole Mediates Proteasomal Degradation of GLI Transcription Factors in Acute Myeloid Leukemia

**DOI:** 10.3390/ijms221910670

**Published:** 2021-10-01

**Authors:** Fabian Freisleben, Franziska Modemann, Jana Muschhammer, Hauke Stamm, Franziska Brauneck, Alexander Krispien, Carsten Bokemeyer, Karl N. Kirschner, Jasmin Wellbrock, Walter Fiedler

**Affiliations:** 1 Department of Oncology, Hematology and Bone Marrow Transplantation with Section Pneumology, Hubertus Wald University Cancer Center, University Medical Center Hamburg-Eppendorf, 20251 Hamburg, Germany; fabian.freisleben@stud.uke.uni-hamburg.de (F.F.); f.modemann@uke.de (F.M.); j.muschhammer@uke.de (J.M.); hauke.stamm@astrazeneca.com (H.S.); f.brauneck@uke.de (F.B.); alexander.krispien@stud.uke.uni-hamburg.de (A.K.); c.bokemeyer@uke.de (C.B.); j.wellbrock@uke.de (J.W.); 2 Mildred Scheel Cancer Career Center, University Cancer Center Hamburg, 20251 Hamburg, Germany; 3 Department of Computer Science, University of Applied Sciences Bonn-Rhein-Sieg, 53757 Sankt Augustin, Germany; karl.kirschner@h-brs.de

**Keywords:** GLI, AML, MBZ, mebendazole, HSP90, HSP70

## Abstract

The prognosis of elderly AML patients is still poor due to chemotherapy resistance. The Hedgehog (HH) pathway is important for leukemic transformation because of aberrant activation of GLI transcription factors. MBZ is a well-tolerated anthelmintic that exhibits strong antitumor effects. Herein, we show that MBZ induced strong, dose-dependent anti-leukemic effects on AML cells, including the sensitization of AML cells to chemotherapy with cytarabine. MBZ strongly reduced intracellular protein levels of GLI1/GLI2 transcription factors. Consequently, MBZ reduced the GLI promoter activity as observed in luciferase-based reporter assays in AML cell lines. Further analysis revealed that MBZ mediates its anti-leukemic effects by promoting the proteasomal degradation of GLI transcription factors via inhibition of HSP70/90 chaperone activity. Extensive molecular dynamics simulations were performed on the MBZ-HSP90 complex, showing a stable binding interaction at the ATP binding site. Importantly, two patients with refractory AML were treated with MBZ in an off-label setting and MBZ effectively reduced the GLI signaling activity in a modified plasma inhibitory assay, resulting in a decrease in peripheral blood blast counts in one patient. Our data prove that MBZ is an effective GLI inhibitor that should be evaluated in combination to conventional chemotherapy in the clinical setting.

## 1. Introduction

The Hedgehog (HH) signaling pathway is a highly conserved signaling cascade that plays a critical role during embryogenesis and is strongly involved in many basic cellular functions, including cell differentiation, proliferation and stem cell maintenance [1]. The main receptor for HH ligands is Patched (Ptch), a 12-pass transmembrane protein. Upon ligand binding Ptch releases SMO, a seven-transmembrane domain G-protein coupled receptor-like protein, which then activates the GLI transcription factors representing the main effectors of the HH signaling pathway. In addition to this canonical HH pathway, numerous signaling cascades result in non-canonical activation of the GLI transcription factors, including *FLT3/STAT5*, *RTK/RAF/MEK/ERK* and *PI3K/AKT/mTOR* [2,3].

It is well established that aberrant activation of HH signaling is associated with a wide variety of neoplasms [4]. Activated GLI transcription factors drive a transcriptional program that promotes survival, growth, migration and stemness [2,4,5]. Expression of GLI1 is associated with a poor prognosis in a wide variety of cancers [6,7]. Moreover, GLI transcription factors play a fundamental role in the maintenance of leukemia, initiating cells that are responsible for therapy failure and tumor relapse due to their chemotherapy resistance [2]. In a previous work, we showed that a high GLI1 and GLI2 expression represents a negative prognostic marker in AML, and that targeted inhibition of GLI1 and GLI2 mediates anti-leukemic effects in vitro and in vivo [7].

Current treatment strategies aim to inhibit GLI signaling by targeting SMO in cancer cells. SMO inhibitors have been tested in AML, where Glasdegib is an approved treatment in conjunction with low-dose cytarabine [8]. However, due to the frequent non-canonical activation of the HH pathway, the inhibition of GLI transcription factors may represent a better choice.

For decades the synthetic benzimidazole Mebendazole (MBZ) has been an approved anthelminthic drug, effective against a broad spectrum of intestinal helminthiasis with a favorable toxicity profile. Indications include short-term and low-dose treatments, as well as high-dose long-term treatments (e.g., 50 mg/kg bodyweight for several months) [9,10]. Besides its anthelmintic activity, MBZ exhibits strong anti-tumor effects in different cancer entities [9]. MBZ’s mechanisms of action are manifold—including anti-angiogenic properties, and inhibition of microtubule depolymerisation and signaling cascades (e.g., BRAF, MEK) [9]. Walf-Vorderwülbecke et al. proposed that MBZ induced c-MYB degradation by inhibiting protein folding through blockade of HSP70 in AML [11]. Herein, we show that MBZ mediates strong anti-leukemic effects by promoting the degradation of GLI transcription factors through inhibition of HSP70/90 chaperone activity, and that MBZ sensitizes AML cells to chemotherapy. Furthermore, two patients with refractory AML were treated with MBZ in an off-label setting, and the clinically achievable MBZ plasma concentrations effectively reduced the GLI signaling activity in a modified plasma inhibitory assay. Our data prove that MBZ is an effective GLI inhibitor that should be evaluated in combination to conventional chemotherapy in the clinical setting.

## 2. Results

### 2.1. MBZ Inhibits SMO Independent Non-Canonical GLI Signaling Predominant in AML

**Figure 1:**
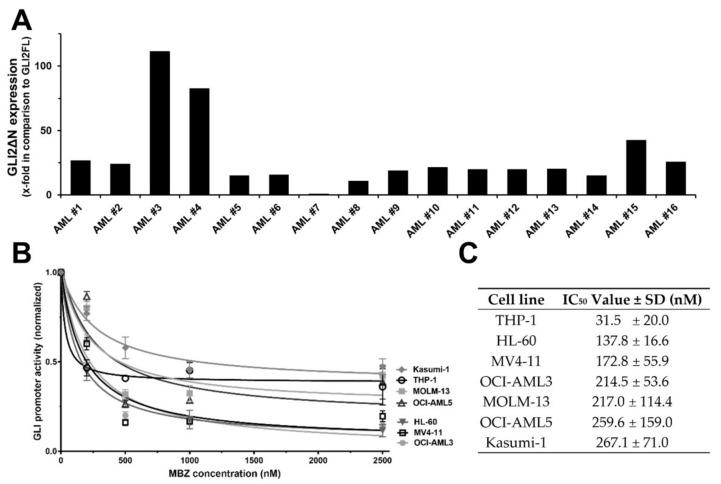
MBZ inhibits SMO independent non-canonical GLI signaling predominant in AML. (**A**) Pretreatment samples of 16 different AML patients with detectable GLI2 expression were analyzed for *GLI2FL* and *GLI2ΔN* expression using RT-qPCR. *GLI2ΔN* expression was normalized to *GLI2FL* expression. (**B**) The AML reporter cell lines MV4-11, MOLM-13, HL60, THP-, Kasumi-1, OCI-AML3 and OCI-AML5 were treated with increasing MBZ concentrations or DMSO as solvent control. The GLI promoter activity was measured after 48 h. (**C**) The IC50 of MBZ on the GLI promoter activity of the different GLI reporter cell lines was calculated from the data shown in (**B**) using a nonlinear regression (curve fit) for inhibitor vs. response with three parameters in GraphPad Prism 7.04.

**Figure 2:**
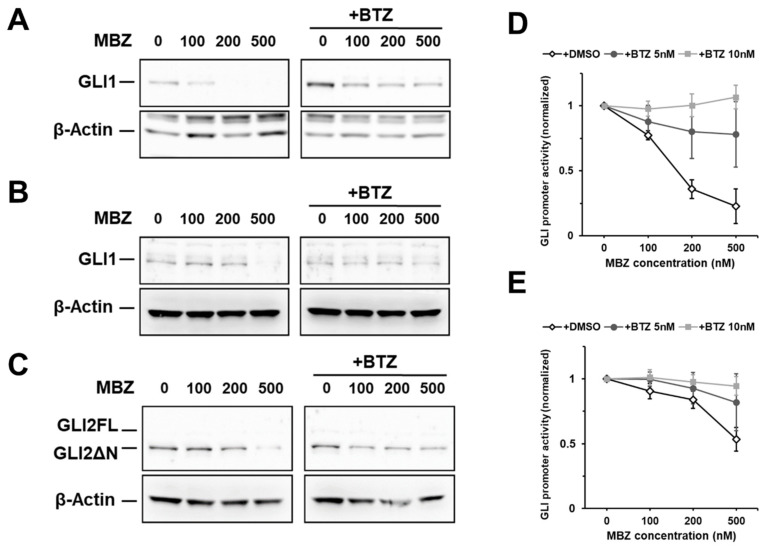
Decreased GLI1 and GLI2FL/GLI2ΔN protein levels upon MBZ treatment. Cell lines THP-1 (**A**) and OCI-AML3 (**B**,**C**) were treated with the indicated concentrations of MBZ alone or in combination with 10 nM BTZ for 24 h. GLI1 (**A**,**B**) and GLI2 (**C**) protein expression was examined by western blot analysis. THP-1 (**D**) and OCI-AML3 (**E**) GLI luciferase reporter cells were treated with MBZ alone or in combination with 5 nM or 10 nM BTZ. The GLI promoter activity was measured after 24 h. Error bars represent the mean values ± standard deviation from at least three independent experiments.

**Figure 3:**
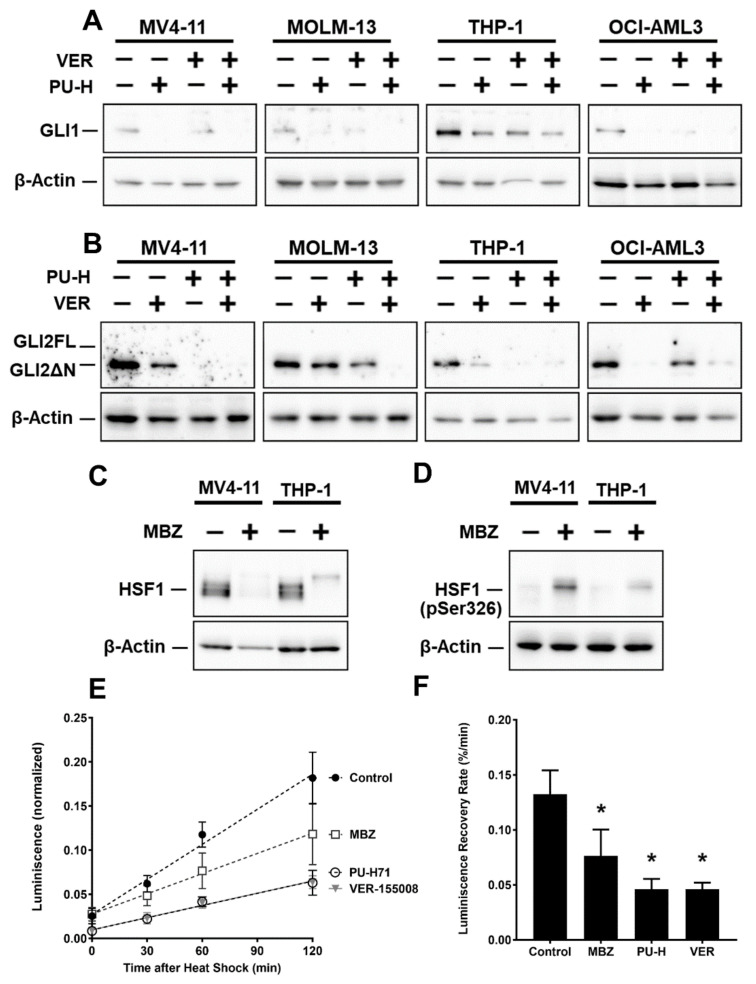
MBZ promotes degradation of GLI Transcription Factors via Inhibition of HSP70/90-Chaperone Activity. AML cell lines MV4-11, MOLM-13, THP-1 and OCI-AML3 have been treated with 1 µM PU-H71 (PU-H) and 25 µM VER-155008 (VER) for 24 h. Subsequently expression of GLI1 (**A**) and GLI2 (**B**) was examined by western blot analysis. (**C**,**D**) AML cell lines MV4-11 and THP-1 were treated with 500 nM MBZ or DMSO as a solvent control for 24 h and protein levels of HSF-1 and phosphorylated HSF-1 (Ser-326) were determined by western blot analysis. Phosphorylated HSF-1 (Ser-326) represents the active state of HSF-1. (**E**) MOLM-13^luc+^ cell line constitutively expressing a firefly luciferase were incubated with 10 µM MBZ, 1 µM PU-H71 (PU-H), 25 µM VER-155008 (VER) or a solvent control. Following a heat-shock, recovery of the intracellular luciferase activity was measured on different time points and normalized to the luciferase activity of the non-heat-shock native control. (**F**) The luminescence recovery rate derived from (**E**) as the slope of the luminescence increases after treatment with MBZ, PU-H71 (PU-H), VER-155008 (VER) or a solvent control. Error bars represent the mean values ± standard deviation from at least three independent experiments. * *p* < 0.05 in the Welch’s *t*-test.

Since the 1987 discovery of GLI1 in human glioma cells [12], the role of the three members GLI1, GLI2 and GLI3 in a variety of cancers has become increasingly apparent [4], with GLI1 expression specifically identified as a negative prognostic factor in numerous cancers [6,7]. Previously, we demonstrated that the treatment of GLI reporter AML cell lines with SMO-inhibitor cyclopamine did not lead to a reduction in GLI promoter activity [3]. We hypothesized that this might be due to the predominant expression of the GLI2ΔN isoform in AML cells. GLI2ΔN represents a constitutively active GLI2 isoform that lacks the amino-terminal repressor domain [13] and has the ability to induce target genes several fold stronger in comparison to the GLI2 full length (GLI2FL) [14]. Expression of GLI2ΔN results in a constitutively active GLI signaling cascade even in the presence of SMO inhibitors, providing an important mechanism for resistance to SMO inhibitors in cancer [15]. Consequently, we analyzed the expression of GLI2ΔN and GLI2FL in samples from 47 newly diagnosed AML patients by qPCR. GLI2 expression was detected in 16 of the 47 samples (34%). *GLI2ΔN* mRNA expression was 29.5-fold higher than the expression of *GLI2FL* mRNA (with a range of 0.8- to 111.5-fold; Figure 1A). Moreover, protein levels of GLI2ΔN were considerably higher than those of GLI2FL in the AML cell lines used herein as determined by western blot (Figure 2C, Figure 3B, Supplementary Materials Figure S1I). This indicates that GLI2ΔN is the predominantly expressed isoform, relative to GLI2FL, in AML.

The anthelmintic MBZ has shown to exhibit strong anti-tumor effects in preclinical studies [9]. In order to investigate if MBZ inhibits the GLI cascade, we treated seven AML GLI reporter cell lines with increasing MBZ concentrations for 48 h. Endogenous expression of GLI transcription factors has been detected in leukemic blasts from a large proportion of AML patients and cell lines [7,16] and was also found in all AML cell lines used herein. Treatment with MBZ led to a strong dose-dependent reduction in all analyzed AML reporter cell lines (Figure 1C). The MBZ concentration required to inhibit the GLI promoter activity was within clinically achievable concentrations, with an IC50 ranging from 32 ± 20 to 267 ± 71 nM after 48 h in the AML cell lines tested (Figure 1C). In contrast to MBZ, the active metabolite of albendazole (i.e., Albendazole sulfoxide (ABZ-S)), a closely related benzimidazole-derived anthelmintic agent [17], had no effect on the GLI reporter activity (Supplementary Materials, Figure S2).

### 2.2. MBZ Promotes Proteasomal Degradation of GLI

To analyze the effects of MBZ on GLI expression, AML cell lines MV4-11, MOLM-13, THP-1 and OCI-AML3 were treated with MBZ in increasing concentrations from 100 nM to 500 nM, followed by western blot and RT-qPCR analyses. We found that GLI1 and GLI2 protein levels were clearly reduced in 24 h after MBZ exposure (Figure 2A–C), whereas *GLI1* and *GLI2* mRNA levels did not decrease (Supplementary Materials Figure S1A–H). Incubation of AML cells with MBZ for 48 h strongly affected GLI2ΔN protein levels and thus could overcome SMO inhibitor resistance (Supplementary Materials Figure S1I).

We hypothesized that MBZ decreases the GLI protein levels by promoting their proteasomal degradation. Therefore, we evaluated the influence of the 26S proteasome inhibitor Bortezomib (BTZ) on GLI protein levels and signaling activity after MBZ treatment. AML GLI reporter cell lines THP-1 and OCI-AML3 were treated with 5 or 10 nM BTZ for 24 h. As anticipated, MBZ inhibited GLI signaling activity in a dose-dependent manner. However, BTZ fully reversed MBZ-mediated inhibition of the GLI promoter activity (Figure 2D,E). Consistent with these results, 10 nM BTZ abolished MBZ’s effect on GLI1 and GLI2 protein levels in THP-1 and OCI-AML3 cells in western blot analysis (Figure 2A–C). Taken together, these results strongly suggest that MBZ mediates proteasomal degradation of GLI1 and GLI2.

### 2.3. MBZ Promotes Degradation of GLI Transcription Factors via Inhibition of HSP70/90-Chaperone Activity

The heat shock proteins 70 (HSP70) and 90 (HSP90) tightly cooperate in the protein stabilization of a wide spectrum of client substrates, including transcription factors. Inhibition of either HSP70 or HSP90 disrupts this chaperone machinery and leaves a client protein prone to misfolding, resulting in its ubiquitination and proteasomal degradation. Walf-Vorderwülbecke et al. reported that MBZ promotes proteasomal degradation of transcription factor c-MYB by interaction with HSP70 [11]. However, GLI protein stability has never been associated with heat shock proteins. Inhibition of either HSP70 or HSP90 with small-molecule inhibitors VER-155008 and PU-H71, respectively, resulted in significant reduction in GLI1 and GLI2 protein levels in western blot analysis. Inhibition of both HSP70 and HSP90 by combination of both agents increased the effect considerably (Figure 3A,B). In accordance with the effects mediated by MBZ, GLI1 and GLI2 mRNA levels did not decrease (Supplementary Materials Figure S3).

We also demonstrated that treatment with 500 nM MBZ did not alter HSP70/HSP90 protein expression in MV4-11, MOLM-13, THP-1 and OCI-AML3 after 24 h using western blot analysis (Supplementary Materials Figure S4). However, HSF-1, the major transcription factor of the heat shock response genes, was heavily phosphorylated on Serine 326 in MBZ-treated MV4-11 and THP-1 cells—reflecting an active state of HSF-1 (Figure 3C). Interestingly, the total HSF-1 protein levels were reduced by MBZ treatment compared to control (Figure 3D).

To further investigate if MBZ directly inhibits the enzymatic activity of the HSP chaperone machinery in AML cells, we generated a MOLM-13 cell line constitutively expressing a firefly luciferase (MOLM-13^luc+^). Following heat-shock, refolding of heat-denatured firefly luciferase depends on cooperative chaperone activity of both HSP70 and HSP90. We treated MOLM-13^luc+^ cells with 10 µM MBZ, 25 µM VER-155008, 1 µM PU-H71 or DMSO as a solvent control. The luciferase signal was recovered without an inhibitor in MOLM-13^luc+^ following a heat-shock, but incubation of AML cells with MBZ or specific HSP inhibitors significantly impaired recovery of the signal (Figure 3E,F), suggesting a direct inhibition of HSP70/HSP90-mediated luciferase refolding.

### 2.4. In Silico Modeling of MBZ Bound to HSP90

**Figure 4:**
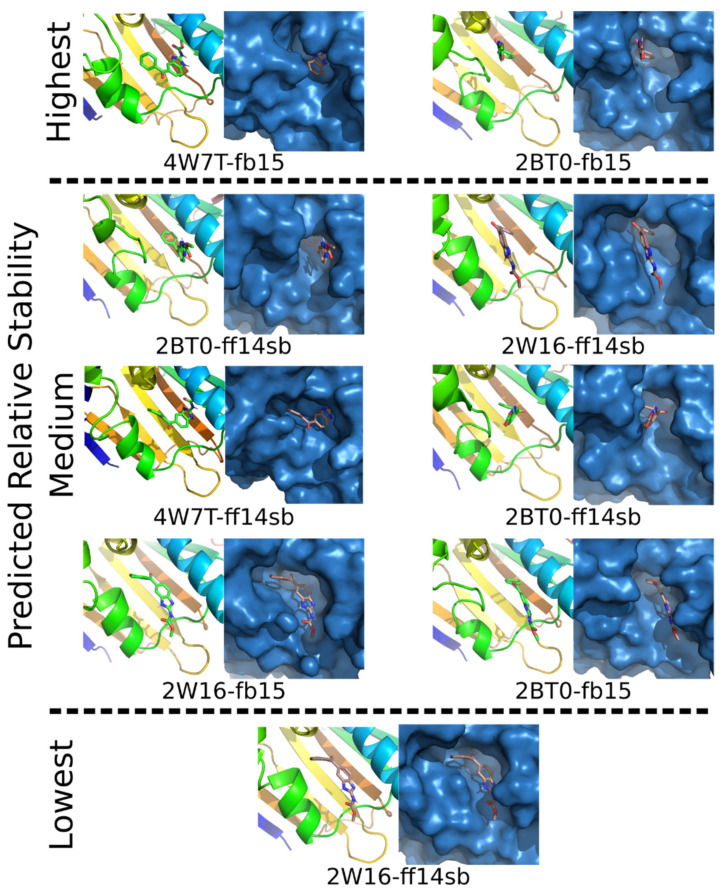
The nine binding poses of MBZ bound to HSP90‘s ATP binding site as identified through hierarchical clustering of the molecular dynamics simulations (600 ns in total). Each pose is shown with the protein rendered as a cartoon and surface. The poses are grouped according to the results of MM/PBSA calculations, with the highest (i.e., 0.0–0.3 kcal/mol), medium (4.6–8.9 kcal/mol) and lowest (12.6 kcal/mol) relative stabilities group together. The source of the poses are noted according to the protein crystal structure and force field used in the modeling. Only the poses identified with populations of >10% in the simulations are shown. Note that clustering a single model’s data can yield multiple poses.

Six molecular models were created to explore how MBZ might bind to HSP90 based on the inhibition data shown above. Using three different HSP90 protein crystal structures to diversify the starting coordinates, MBZ was placed into their ATP binding site. MD simulations were subsequently performed under physiological conditions, allowing MBZ to sample different interactions within the binding pocket (Figure 4). A total of nine poses were identified with distinct orientations and significant populations (Figure 4). Throughout all simulations MBZ remained in the ATP binding site, forming nonbonded interactions with 10-16 amino acids, with the residues Asn51, Ala55, Ile96, Gly97, Met98, Asn106, Leu107, Phe138, Thr184 and Val186 being most frequently involved. A free energy analysis revealed two poses that are mostly likely for being experimentally observed. An extensive analysis of the nine binding poses can be found in reference [18] Manuscript is in preparation, which includes an examination of water involvement, how MZB binding effects amino acid motion and a thorough quantum mechanical study of the possible conformations that MBZ can adopt.

### 2.5. Mebendazole and the GLI Inhibitor GANT-61 Exhibit Synergistic Anti-Leukemic Effects

**Figure 5:**
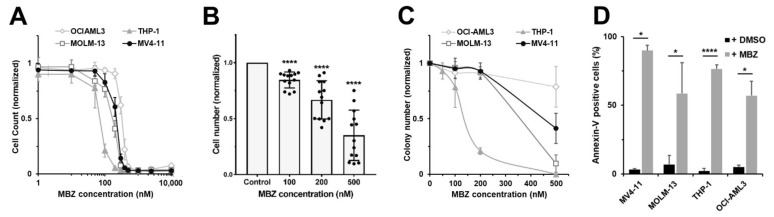
MBZ induces apoptosis and growth inhibition of AML cells. (**A**) Effects of increasing MBZ doses on AML cell lines MV4-11, MOLM-13, THP-1, and OCI-AML3 were analyzed for proliferation after three days. (**B**) Cell growth of primary AML cells (*n* = 13) was determined after 7 days of MBZ treatment. (**C**) Clonogenicity of AML cell lines MV4-11, MOLM-13 and THP-1 with increasing MBZ doses was investigated in colony formation assays. (**D**) Apoptosis induction was measured at 48 h after MBZ treatment in AML cell lines. Error bars represent the mean values ± standard deviation. * *p* < 0.05, **** *p* < 0.0001 in the Welch’s *t*-test.

We treated AML cell lines and primary AML samples with different MBZ concentrations, resulting in a dose-dependent effect on the proliferation, colony formation and apoptosis (Figure 5A–D).

**Figure 6:**
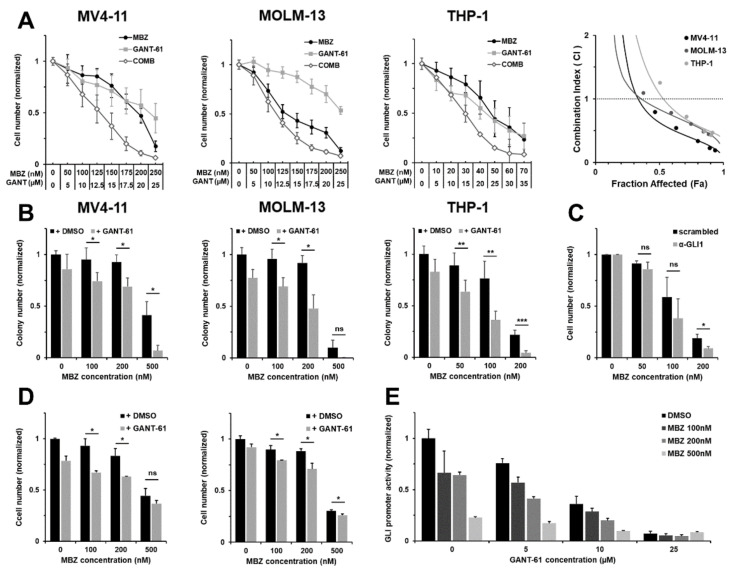
MBZ shows synergistic anti-leukemic effects upon combined treatment with GANT-61. (**A**) MV4-11, MOLM-13 and THP-1 cells were treated with the indicated concentrations of MBZ and GANT-61 alone or in combination and the effect on the proliferation capacity was analyzed after three days. The drug combination was tested for synergy by the method of Chou and Talalay using CompuSyn software. (**B**) The clonogenicity of AML cell lines MV4-11, MOLM-13 and THP-1 under MBZ treatment alone and in combination with GANT-61 was investigated in colony formation assays. (**C**) Effects of MBZ on cell growth of THP-1 cells transduced with shRNA against *GLI1* were analyzed in comparison with control cells transduced with a scrambled shRNA cell growth after 3 days. (**D**) Primary AML cells were treated with MBZ and GANT-61 alone, in combination or a solvent control. The effect on the proliferation capacity was analyzed after three days. Two representative data sets shown out of four AML samples. (**E**) GLI Reporter AML cell line THP-1 was treated with MBZ and GANT-61 alone or in combination. The GLI promoter activity was measured after 24 h. Error bars represent the mean values ± standard deviation. * *p* < 0.05, ** *p* < 0.01 in the Welch’s *t*-test.

To evaluate the anti-leukemic activity of MBZ upon combined inhibition of GLI, we also investigated its usage with the small molecule GLI inhibitor GANT-61. We treated the AML cell lines MV4-11, MOLM-13, THP-1 and OCI-AML3 with combinations of MBZ and GANT-61, and analyzed cell proliferation and colony formation. In all cell lines tested, MBZ treatment alone already resulted in decreased proliferation and colony forming capacity in a dose dependent manner (Figure 5A–C). The combination of MBZ with the GLI inhibitor GANT-61 synergistically increased MBZ’s anti-proliferative effects on all three AML cell lines (Figure 6A). Therapeutic synergy between MBZ and GANT-61 was indicated by a combination index < 1 calculated using the Chou-Talalay-Method (Figure 6A). In colony formation assays, treatment with high MBZ concentrations, in particular, resulted in significant reduction in colony numbers of MV4-11, MOLM-13 and THP-1 cells. Furthermore, GLI inhibition by GANT-61 increased the effect of MBZ on colony formation significantly (Figure 6B). Moreover, inhibition of HH signaling using shRNA targeting *GLI1* sensitized THP-1 cells to anti-proliferative effects by MBZ compared to control cells transduced with a scrambled shRNA (Figure 6C). Freshly isolated primary AML cells from 13 patients were investigated for anti-proliferative effects of MBZ treatment alone and in combination with GANT-61. MBZ mediated a strong and significant inhibitory impact on primary AML cell growth (Figure 5A), which was further increased by combination with GANT-61 (Figure 6D). Additionally, we treated the GLI luciferase reporter AML cell line THP-1 with MBZ or GANT-61 alone and in combination for 24 h and measured the GLI promoter activity. As expected, MBZ and GANT-61 reduced the GLI promoter activity compared to the untreated control. The inhibitor combination resulted in a more pronounced decrease relative to the single agent treatment (Figure 6E).

### 2.6. MBZ Sensitizes AML Cells to Chemotherapy

**Figure 7:**
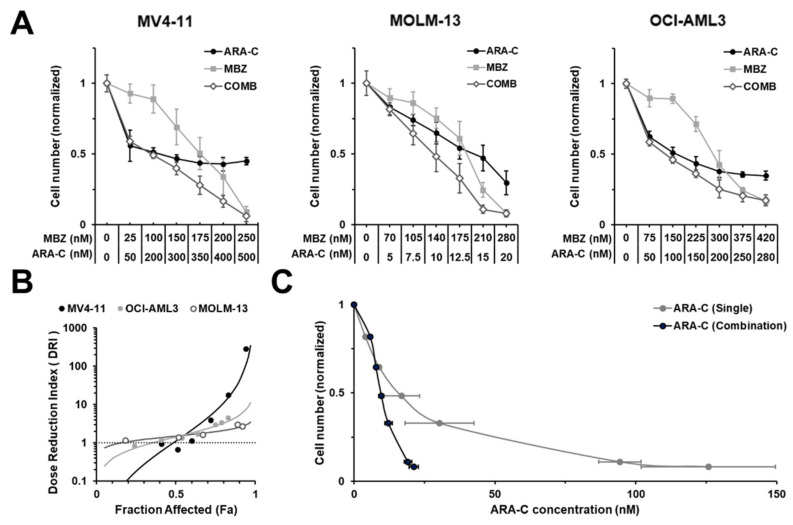
MBZ significantly sensitizes AML cells to cytarabine. (**A**) AML cell lines MV4-11, MOLM-13 and OCI-AML3 were treated with the indicated concentrations of MBZ and cytarabine (ARA-C) alone or in combination and the effect on the proliferation capacity was analyzed after three days. (**B**) Dose reduction index (DRI) of MBZ and GANT-61 combination on proliferation of MV4-11, MOLM-13 and OCI-AML3 cells. Values have been calculated from data of at least three independent experiments (**A**) using CompuSyn Software for Drug Combinations. DRI > 1 indicating a favorable drug combination. (**C**) Effect-dose-relationship of cytarabine (ARA-C) on proliferation of MOLM-13 cells alone or in combination with MBZ, respectively. Values have been calculated from data of at least three independent experiments using CompuSyn Software for Drug Combinations. Error bars represent the mean values ± standard deviation.

Cumulating evidence suggests that active GLI signaling plays a fundamental role in the maintenance of leukemia initiating cells, which evade chemotherapy due to their high drug resistance against cytotoxic drugs [19]. Consequently, they are associated with residual disease, relapse and therapy failure. Therefore, we examined the combinational effect of cytarabine and MBZ on the cell growth of AML cell lines MV4-11, MOLM-13 and OCI-AML3. As shown in Figure 7A, combined effect of cytarabine and MBZ induced a significant reduction in cell growth compared to each agent alone. To quantify if the combination of MBZ and cytarabine represents a favorable drug combination, data were analyzed using CompuSyn to compute the dose-reduction index (DRI) values for the drug combination tested at a constant dose ratio. DRI values represent the fold decrease in the dose of a drug needed in a combination to achieve the same efficacy as the drug alone. DRI values  > 1 are considered favorable with regard to the predicted reduction in toxicity of a drug therapy. These parameters are particularly relevant for the analysis of drug combinations in the context of cancer treatment. When combined with MBZ, cytarabine doses can be reduced by 288.0-, 2.7- and 4.5-fold in MV4-11, MOLM-13 or OCI-AML3, respectively, to meet the same anti-proliferative effect level (Figure 7B). In all three cell lines, the DRI values increased with the rising effect level (Fraction affected (Fa)), suggesting the beneficial effect is particularly pronounced in the relevant effect level of a cancer therapy (Figure 7B,C).

### 2.7. MBZ Effectively Inhibits GLI Signaling in Clinically Achievable Plasma Levels

**Figure 8:**
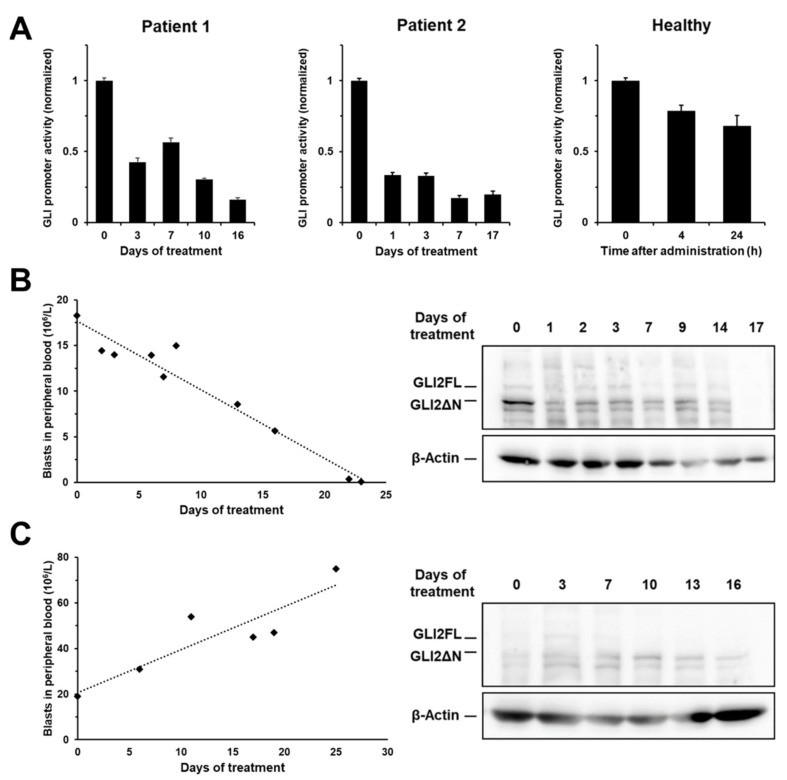
Clinically achievable plasma concentrations of MBZ effectively inhibit GLI signaling. (**A**) Two patients with refractory AML were treated with MBZ monotherapy with a daily dose of up to 50 mg/kg. Patient blood samples taken at different time points over the period of 16 to 17 days. Isolated plasma samples were incubated GLI luciferase reporter cell line OCI-AML3 for 24 h before GLI promoter activity was measured. A healthy volunteer ingested MBZ at a dose of 50 mg/kg divided into two doses at time 0 h and 12 h. Blood was drawn at 4 h and at 24 h. In a modified Plasma Inhibitory Assay (PIA) MV4-11 GLI luciferase reporter cells were incubated with isolated plasma samples and GLI promoter activity measured after 24 h using the Dual-GLO Luciferase Assay Kit and the Infinite F200 PRO reader. Patient 1 (**B**) and patient 2 (**C**) with refractory AML were treated with MBZ monotherapy as described above. Under MBZ therapy, the blast counts in the peripheral blood of the AML patients were measured. Peripheral mononuclear cells (PBMC) of patient 1 and patient 2 were isolated from several samples throughout the entire treatment period and GLI2 analyzed by western blot analysis. β-Actin was used as a loading control. Error bars represent the mean values ± standard deviation.

To further evaluate if MBZ is a suitable therapeutic agent to inhibit GLI, we transferred these findings into the clinical setting by treating two refractory AML patients with MBZ monotherapy in an off-label setting. Using a modified plasma inhibitory assay (PIA) by incubating an indicator cell line carrying the GLI luciferase promoter transgene with the patients’ plasma, a reduction in GLI promoter activity was detected for both samples (Figure 8A). Moreover, a 62-year-old male healthy volunteer ingested MBZ at a dose of 50 mg/kg divided over two ingestions at time 0 h and 12 h. Blood was drawn at 4 h and at 24 h. PIA results indicated a biological active plasma concentration (Figure 8A). Two patients with refractory AML received MBZ monotherapy after informed consent: Patient 1, a 66-year-old female, with normal karyotype and *NPM1*, *FLT3-TKD* and *IDH1* mutations (ELN favorable risk). This patient had received 2 induction cycles of cytarabine and daunorubicine followed by three consolidation therapies with cytarabine and in relapsed setting, mitoxantrone, cytarabine and etoposide (MEC) with no response to treatment); Patient 2, a 74-year-old female, had adverse risk (ELN criteria) secondary AML after MDS according with a complex aberrant karyotype (deletion 5, deletion 8 and monosomy 17 with no additional AML specific mutations). This patient has been treated non-intensively with low-dose cytarabine and venetoclax with no response to therapy prior to MBZ treatment. In patient 1, a clear and continuous decrease in leukemic blasts in peripheral blood and a fast reduction in GLI2 levels in peripheral leukemic blood cells could be shown whereas patient 2 did not respond. (Figure 8B,C).

## 3. Discussion

MBZ is a broad spectrum benzimidazole used for several decades in human and veterinary medicine to treat a variety of parasitic worm infections [17]. Lately, MBZ gained attention as a promising candidate for drug repurposing in oncology due to multiple studies reporting substantial in vitro and in vivo anticancer effects [9]. Besides neuroblastoma, hematological malignancies—including leukemia, lymphoma and multiple myeloma—were identified as the most sensitive cancers to treatment with the MBZ analogue flubendazole, as shown in a screen of 321 cell lines from 26 cancer entities [20].

In this study, the anti-leukemic effects of MBZ were confirmed in AML cell lines and primary blasts from AML patients. Most importantly, we revealed that MBZ’s anti-leukemic effects were, at a minimum, partly due to a significantly reduced activity of the HH transcription factors GLI1 and GLI2 in AML. Furthermore, our data strongly indicate that MBZ mediates its anti-leukemic effects by promoting the degradation of GLI transcription factors through inhibition of HSP70/90 chaperone activity. Interestingly, MBZ sensitized AML cells to chemotherapy.

We previously demonstrated the importance of GLI1 and GLI2 in AML pathophysiology [7], and showed that inhibition of GLI activity resulted in pronounced anti-leukemic effects in vitro and significantly prolonged survival in a leukemic mouse model. We also showed that high expression of GLI represents a negative prognostic factor in two independent AML patient cohorts [7]. The potent anti-cancer effects mediated by inhibition of GLI transcription factors have also been demonstrated in numerous other studies [21].

Previously, Larsen et al. showed that MBZ inhibits canonical HH signaling in Shh Light2 fibroblasts by inhibiting the formation of the primary cilium [22], which is required for SMO-mediated GLI activation [23]. However, in previous work we could show that the treatment with the SMO inhibitor cyclopamine had no impact on the GLI promoter activity in AML reporter cell lines—leading to the hypothesis that the activation of GLI proteins in AML cells occurs independently of SMO [3]. In line with this theory, Chaudhry et al. also demonstrated that GLI signaling occurs independently of SMO [16]. Another study showed that primary cilia, which are essential for functional SMO signaling, are absent in most AML cells [24]. Mounting evidence implicates SMO-independent, non-canonical ways of GLI activation by alternative oncogenic pathways in AML, including *FLT3-ITD*, *PI3K/AKT/mTOR* and *RAS/RAF/MEK/ERK* signaling cascades [2,3]. These results support our hypothesis that MBZ mediates its inhibitory potential against the HH pathway in an SMO-independent way by inhibiting GLI downstream from SMO. 

We could show that the decrease in GLI promoter activity resulted from a reduction in GLI1 and GLI2 protein levels. Inhibition of the 26S proteasome by Bortezomib abolished the effect of MBZ on GLI protein levels, indicating that the reduced GLI protein levels are caused by degradation via this proteasome.

Heat shock proteins act as molecular chaperones that are involved in the folding, activation and assembly of a variety of proteins. HSP70 and HSP90 are believed to act as the core chaperone system regulating stability, trafficking and degradation of signaling proteins [25,26] and therefore maintaining the activity of a variety of protein kinases, transcription factors and steroid hormone receptors [26]. Based on our hypothesis that MBZ promotes the proteasomal degradation of GLI transcription factors, we investigated the effect of inhibition of HSP70 and HSP90 on GLI transcription factors. Treatment of AML cells with small molecule HSP70 and HSP90 inhibitors resulted in a marked decrease in GLI1 and GLI2 protein levels without reducing mRNA levels. Walf-Vorderwülbecke et al. extensively demonstrated the ability of MBZ to inhibit HSP70 [11]. However, such an effect of MBZ on HSP90 has not yet been demonstrated. Based on the strong effects of MBZ on GLI1 and GLI2 protein levels, which were comparable to dual inhibition with an HSP90 and an HSP70 inhibitor in our experiments, we hypothesized that MBZ might be an inhibitor of HSP70 and HSP90. To further support this hypothesis, in silico molecular models were created by binding MBZ to the ATP binding site. The modeling predicts that MBZ forms short-range nonbonded interaction with at least 10–16 amino acids within the binding site. Thus, the combined experimental and theoretical data strongly support the idea that MBZ binds to heat shock proteins and inhibits their binding to other proteins.

For binding to HSP90, its client proteins require other chaperones and co-chaperones since they are unable to be bound by HSP90 directly. Certain client proteins, such as transcription factors, have to be bound by HSP70 and its co-chaperone HSP40 first before being delivered to HSP90 [26]. Disruption of the HSP70-HSP90 chaperone cascade results in misfolding and degradation of those client proteins [27]. The strong sensitivity to inhibition of HSP70 and HSP90 in our study suggests that GLI transcription factors rely heavily on the HSP70-HSP90 chaperone cascade for protein folding and stability [28].

Similar to inhibitors of HSP70 or HSP90, MBZ was able to inhibit refolding of heat-denatured luciferase in MOLM^luc+^ AML cells. This suggests that MBZ mediates its effect, at least in part, by disrupting the cellular protein folding machinery. In line with our findings, Walf-Vorderwülbecke et al. demonstrated that MBZ is able to interfere with different members of the HSP70-HSP90 chaperone family in AML cells using nematic protein organization technique (NPOT^®^) analysis and DAVID analysis [11]. They indeed showed that the association of c-MYB with the HSP70 complex was lost after MBZ exposure, leading to misfolding and subsequent proteasomal degradation of MYB [11].

Treatment of AML cell lines with MBZ had no effect on the HSP70 or HSP90 protein expression, as shown by western blot analysis. However, HSF-1, the major transcription factor of the heat shock response genes, was heavily phosphorylated at Serine 326 in MBZ-treated AML cells, a modification that is critical for stress-induced HSF-1 activation [29]. Triggering of the HSF-1 stress response by MBZ could be due to proteotoxic stress that is also caused by other HSP inhibitors [29]. On the other hand, MBZ induced a reduction in total HSF-1 protein levels in AML cells. A possible explanation might be the depletion of factors that stabilize HSF-1 protein levels, such as Bcl-2 interacting cell death suppressor (BIS) [27,30,31,32].

It should be noted that for MBZ—in addition to the inhibition of HSP70 and HSP90, and subsequent degradation of transcription factors (e.g., GLI1, GLI2, MYB) and other HSP client proteins (e.g., FLT3)—a variety of other mechanisms have been identified that can mediate both inhibitory effects on GLI signaling and generate anticancer effects. Observed MBZ-mediated anticancer effects include the induction of anti-tumor immune response, sensitization to radiation and chemotherapy, inhibition of angiogenesis, induction of apoptosis, and inhibition of proliferation [9]. For instance, MBZ was shown to inhibit several important signaling kinases, including VEGFR2, FAK, GTPases Rho-A and Rac1 [9], ABL, JNK3 and KIT [33]. Furthermore, it was revealed that MBZ inhibits BRAF and MEK by blocking their ATP binding pocket [34]. Furthermore, MBZ inhibits tubulin depolymerization in several tumor models, including non-small cell lung cancer and glioblastoma [35,36]. However, in AML, MBZ did not induce microtubule depolymerization in concentrations of up to 10 µM [11].

We could show that in AML cells, the combination of MBZ with the small-molecule GLI inhibitor GANT-61 leads to synergistic anti-leukemic effects. This could be used as a potential treatment strategy in the future to enhance the efficacy of a pharmacological HH blockade. Several HH pathway inhibitors are in development for AML treatment and are being considered as a new class of therapeutics [37]. MBZ represents a promising candidate to potentiate the effect of these agents and maximize their therapeutic success. Moreover, MBZ sensitized AML cell lines MV4-11, MOLM-13 and OCI-AML3 to cytarabine as indicated by large positive DRI values. This possible dose reduction could lower the therapy’s toxicity while maintaining the same anti-leukemic effect. Consequently, this would be a suitable approach to achieve an improved treatment for the elderly who cannot tolerate higher doses. [38,39]. The prognosis for elderly, unfit patients is still bleak with current treatments. Although, fortunately, in recent years, the prognosis for these patients has brightened-up with the introduction of new targeted agents such as Bcl-2, FLT3 and IDH1/2 inhibitors. Especially the combination of azacitidine and venetoclax has become a wide-spread regimen for elderly, unfit AML patients resulting in an increased overall survival compared to hypomethylating agents alone [40]. However, not all new treatment modalities are curative, and treatment options for patients becoming refractory to these regimens are sparse. Therefore, MBZ may represent a valuable therapeutic option in this setting because of its very favorable toxicity profile, which is well suited for the treatment of elderly patients [38,39].

Long-term, repeated administration of mebendazole results in significantly higher plasma levels compared to a single dose, possibly due to enterohepatic circulation [41]. There is a large interindividual variation in the plasma levels achieved, with one study showing a maximum plasma concentration ranging from 0.017–0.134 µM after a single 1.5 g dose and up to 0.5 µM after repeated administrations of 1 g [42]. In another study, 12 patients with cystic disease were treated with a single or repeated dose of 10 mg/kg. Single dose administration resulted in a maximum plasma concentration of 0.24 µM on average (ranging from 0.06–1.69 µM), while repeated administration resulted in a maximum concentration of 0.47 µM and an Area Under The Curve five times higher than after a single dose [41]. We demonstrated that clinically achievable plasma concentrations of MBZ effectively inhibit GLI signaling in all three subjects (two AML patients and one healthy volunteer). Most notably, one patient with refractory AML treated with MBZ monotherapy in an off-label setting responded with a clear and continuous decrease in leukemic blasts in peripheral blood and a fast reduction in GLI2 levels in peripheral leukemic blood cells. In agreement with previous studies on MBZ plasma levels, repeated administration of the drug resulted in a stronger inhibitory effect than a single dose. Furthermore, and most importantly, MBZ-treatment led to strong anti-leukemic effects in one patient which was consequently accompanied by reduction in GLI2 protein levels in blast lysates. This suggests that oral administration of MBZ is suitable for achieving noticeable therapeutic effects in clinical use.

In summary, our work highlights the exceptional potential of MBZ as a future therapeutic option for the treatment of diverse cancers. Based on the results herein, we are currently pursuing a clinical trial with MBZ and low dose cytarabine for the treatment of elderly, refractory AML patients.

## 4. Materials and Methods

### 4.1. Cell Lines and Cell Culture

Cell lines MV4-11, MOLM-13, HL-60, THP1, Kasumi-1, OCI-AML3 and OCI-AML5 were either purchased at the DSMZ (Deutsche Sammlung von Mikroorganismen und Zellkulturen GmbH, Heidelberg, Germany) or at the ATCC (American Type Culture Collection, Manassas, VA, USA) or authenticated by the Multiplex human Cell Authentication test (Multiplexion, Heidelberg, Germany). MV4-11, MOLM-13, HL-60 and THP1 cells were maintained in RPMI 1640 medium (Gibco, Thermo Fisher Scientific, Waltham, MA, USA) supplemented with 10% fetal bovine serum (FBS Superior, Biochrom GmbH, Berlin, Germany). Kasumi-1 cells were cultured in RPMI 1640 medium supplemented with 20% FBS. OCI-AML3 cells were maintained in α-MEM medium (Gibco, Thermo Fisher Scientific, Waltham, MA, USA) supplemented with 20% FBS. OCI-AML5 cells were cultured in α-MEM medium supplemented with 20% FBS and 10 ng/mL GM-CSF (PeproTech GmbH, Hamburg, Germany). All cells were maintained in a humidified incubator with 5% CO_2_ at 37 °C. Primary AML cells were obtained after patient’s informed consent and approval of the study by the ethics committee (PV3469, Ethik-Kommission der Ärztekammer Hamburg). Cells were isolated from bone marrow using density gradient centrifugation and cultured as described elsewhere [43].

### 4.2. Inhibitors and Reagents

Mebendazole ((Methyl-N-(5-benzoyl-1H-benzimidazol-2-yl)-carbamat, #M2523) and VER-155008 (#SML027) were purchased from Sigma-Aldrich (St. Louis, MO, USA). Cytarabine (Cytosin-1-β-D-arabinofuranosid) was purchased from Cell Pharm GmbH (Ara-Cell, 06983044, STADA, Bad Vilbel, Germany). GANT-61 (2,20-[[Dihydro-2-(4-pyridinyl)-1,3(2H,4H)-pyrimidinediyl]bis(methylene)]bis[N,N-dimethylbenzenamine], #3191) and PU-H71 (6-Amino-8-[(6-iodo-1,3-benzodioxol-5-yl)thio]-N-(1-methylethyl)-9H-purine-9-propanamine, #3104) were purchased from Tocris Biosience (Bristol, UK).

### 4.3. Reverse Transcription and Quantitative PCR

Exon-spanning primers were designed with Primer Blast [44] or Primer3web [45]. RNA was extracted using innuPREP RNA Mini Kit 2.0 (845-KS-2040250, Analytik Jena, Jena, Germany) and reverse transcribed into cDNA using PrimeScript™ RT Master Mix (RR036B, TaKaRa Bio Inc., Kusatsu, Japan). RT-qPCR analyses were carried out on the LightCycler 96 (Roche, Basel, Swiss) using the TB Green Premix Ex Taq II (RR820B, TaKaRa Bio Inc., Kusatsu, Japan) over 40 PCR cycles. Relative expression of the target genes was normalized to the reference gene *glyceraldehyde 3-phosphate dehydrogenase* (*GAPDH*) and calculated using the Pfaffl method [46].

Differentiation of *GLI2* and *GLI2ΔN* was performed according to the method of Londoño et al. [47]. The expression of the full-length transcript (*GLI2-FL*) and the C-terminus (*GLI2-C-term*) were determined in relation to total *GLI2* (*GLI2-ALL*). The expression of *GLI2ΔN* was calculated by subtracting the expression of *GLI2-FL* from the expression of *GLI2-C-term*. The difference corresponds to the expression of *GLI2ΔN*.

Primers are listed in Appendix A (Table A1).

### 4.4. Protein Isolation and Western Blot

Protein isolation and western blots were performed as described before [48]. For analysis of phosphorylated proteins, cells were harvested and lysed with radioimmunoprecipitation assay (RIPA) buffer (89900, Thermo Fisher Scientific, Waltham, MA, USA) supplemented with protease and phosphatase inhibitors (cOmpleteTM Tablets, 11697498001, Roche, Basel, Swiss; Sodium orthovanadate, 13721-39-6, Fivephoton Biochemicals, San Diego, CA, USA). Antibodies against rabbit anti-GLI1 monoclonal antibody (C68H3, 1:1000, Cell Signaling Technology, Inc., Danvers, MA, USA), mouse anti-GLI-2 (Dilution, C-10: sc-271786, Santa Cruz Biotechnology, Dallas, TX, USA), rabbit Anti-HSF1-phospho(S326) antibody (1:5000, EP1713Y, abcam, Cambridge, UK), rabbit Anti-HSF1 antibody (1:1000, 4356, Cell Signaling Technology, Inc., Danvers, MA, USA), Mouse anti-HSP70/HSPA1A antibody (1:1000, MAB1663, R&D Systems, Inc. Minneapolis, MN, USA), Rabbit anti-HSP90 Antibody (1:1000, #4874, Cell Signaling Technology, Inc., Danvers, MA, USA) or mouse anti-β-ACTIN (sc-47778, 1:5000, Santa Cruz Biotechnology, Dallas, TX, USA). Secondary antibodies against HRP-linked anti-rabbit immunoglobulins (1:10,000, 7074) and anti-mouse IgG (1:10,000, NXA931) secondary antibodies were purchased from Cell Signaling Technology (Danvers, MA, USA) and GE Healthcare (Cytiva, Marlborough, MA, USA), respectively.

### 4.5. GLI Reporter Assays

Stable GLI reporter AML cell lines were transduced by lentiviral constructs containing the *firefly luciferase* gene under the control of GLI transcriptional response elements and as internal control the *renilla luciferase* gene under the control of CMV promoter elements (Cignal™ Lenti Reporters, S-6030L, Qiagen, Venlo, The Netherlands) followed by puromycin (P7255, Sigma-Aldrich, St. Louis, MO, USA) and hygromycin (1287.1, Carl Roth GmbH, Karlsruhe, Germany) selection. Stable GLI reporter cells were treated with MBZ, GANT-61, PU-H71, VER-155008 or a solvent control, with the GLI promoter activity measured after 24 h using the Dual-GLO Luciferase Assay Kit (E2940, Promega, Madison, WI, USA) and the Infinite F200 PRO reader (Tecan, Männedorf, Switzerland). The firefly luciferase-mediated GLI promoter activity was normalized to the renilla luciferase-mediated CMV promoter activity.

### 4.6. Plasma Inhibitory Assay

OCI-AML3 reporter cells were plated at a ratio of 1:9 in serum-free medium plus plasma sample and incubated for 24 h at 37 °C. Before measurement, cells were washed three times with serum-free culture medium and the luciferase-mediated GLI promoter activity was measured in triplicates as described above.

### 4.7. Refolding Assay

MOLM-13^luc+^ cells stably expressing *firefly luciferase* were generated by lentiviral transduction using a luciferase-encoding vector kindly provided by Kristoffer Riecken [49]. The 1.5 million MOLM-13^luc+^ cells were incubated with 10 µM MBZ, 1 µM PU-H71, 25 µM VER-155008 or a solvent control at 37 °C for 30 min. Subsequently, cells were exposed to a heat-shock by incubation at 42 °C for 30 min, while the non-heat-shock control was incubated at 37 °C. Following heat-shock, cells were incubated at 37 °C and intracellular luciferase activity was measured on different time points (0, 30, 60, 120 min) using the Dual-GLO Luciferase Reagent (E2940, Promega, Madison, WI, USA) and the Infinite F200 PRO reader (Tecan, Männedorf, Switzerland).

### 4.8. Proliferation Assay

AML cells were incubated with different concentrations of MBZ alone or in combination with either GANT-61 or cytarabine for 3 or 7 days. Viable cell numbers were determined with the Trypan Blue dye exclusion method using the cell viability analyzer Vi-Cell ™ XR (Beckman Coulter, Brea, CA, USA).

### 4.9. Apoptosis Assay

AML cells were incubated with different concentrations of MBZ. Induction of apoptosis was measured after 48 h by flow cytometry using an APC-conjugated Annexin-V (AnxA100, MabTag GmbH, Friesoythe, Germany) and propidium iodide (P3566, Invitrogen, Thermo Fisher, Waltham, MA, USA) Data analysis was performed using the FACS Calibur (BD Biosciences, San Jose, CA, USA) and FlowJo V10 (Version 10.0.7, BD Life Sciences, FlowJo, LLC, Ashland, OR, USA) software.

### 4.10. Colony Formation Assay

AML cell lines were incubated with different concentrations of MBZ and/or GANT-61 and cultured in methylcellulose-based semi-solid media without or supplemented with growth factors, respectively (04230, Methocult H4230, Stemcell Technologies, Vancouver, BC, Canada). After seven days, the number of colonies was counted using an inverted microscope (Axiovert 25, Zeiss, Jena, Germany).

### 4.11. Cloning and Lentiviral Transduction

A pLKO.1-puro vectors encoding *GLI1* (TRCN0000020485, sequence 5′CCGGCCTGATTATCTTCCTTCAGAACTCGAGTTCTGAAGGAAGATAATCAGGTTTTT-3′), or *scrambled* shRNA (SHC002, non-target shRNA vector) were purchased from Sigma-Aldrich (St. Louis, MO, USA). The Lentiviral Gene Ontology Vector (LeGO) system was used for cloning and transfection into the AML cell lines (LeGO-G/Puro) [49]. Lentiviral particle containing supernatants were generated in HEK293T cells co-transfected with the plasmids LeGO-G/Puro (*GLI1* shRNA) or LeGO-G/Puro (*scrambled* shRNA) in combination with pMD2.G-VSV-G and psPAX2-Gag-Pol using calcium phosphate co-precipitation. THP-1 cells were either transduced with non-targeting shRNA (negative control) or with shRNA against *GLI1*. On day 3 after transduction, transduced cells were selected by addition of puromycin (2 μg/mL; Sigma-Aldrich, St. Louis, MO, USA) for 7 days prior to functional assays. The knock-down efficiency for GLI1 was determined using quantitative PCR analysis after seven days of puromycin selection. To generate the MOLM-13^luc+^ cells, MOLM-13 cells were transduced with a LeGO vector encoding for *firefly luciferase* using the same protocols. All work with lentiviral particles was done in a S2 facility after approval according to German law.

### 4.12. In Silico Modeling

All-atom molecular dynamic (MD) simulations were performed on models where MBZ was bound to HSP90′s ATP binding site using the AMBER software package [50]. A total of 6 independent simulations were performed that differed in the protein’s crystal structure used (i.e., 2WI6, 2BT0 and 4W7T [51,52,53]) for model development and in the force field employed during the simulation. The proteins were modeled using the ff14SB and fb15 force fields [54,55], while MBZ was modeled using the gaff2 force field and RESP partial atomic charges [56]. The models were solvated using TIP3P or force-balanced water models, as appropriate for the protein force field. In total, 600 nanoseconds of simulation data were collected and analyzed. A complete description of the modeling can be found in reference [18]. 

### 4.13. Statistical Analysis

Data from the in vitro assays were statistically analyzed by the Welch’s t-test using GraphPad Prism 7 (GraphPad Software, Inc., San Diego, CA). A *p* value less than 0.05 was considered to be statistically significant. The combination index (CI) and dose reduction index (DRI) were calculated using the CompuSyn program (Version 1.0, ComboSyn Inc., Paramus, NJ, USA) based on the Chou Talalay Method [57].

## Supplementary Materials

The following are available online at https://www.mdpi.com/article/10.3390/ijms221910670/s1, Figure S1: Treatment with MBZ does not reduce GLI1 or GLI2 mRNA expression in AML cell lines, Figure S2: Treatment with ABZ does not reduce GLI promoter activity in AML reporter cell lines, Figure S3: Treatment with HSP70 or HSP90 inhibitors does not reduce GLI1 or GLI2 mRNA expression in AML cell lines, Figure S4: Mebendazole treatment does not affect HSP70 or HSP90 protein levels.

## Appendix A

**Table A1:** Primers used in this study.

Gene	Sense	Anti-Sense
*GLI1*	CTACATCAACTCCGGCCAAT	CGGCTGACAGTATAGGCAGA
*GLI2*	CCCCTACCGATTGACATGCG	GAAAGCCGGATCAAGGAGATG
*GLI2-ALL*	AACCCTGTCGCCATTCACAA	CCAGTGGCAGTTGGTCTCAT
*GLI2-FL*	TCAGCCTTTGGACACACACC	TGCACTTGTGGGGCTTCTC
*GLI2-C-term*	GCTGCAACAAAGCCTTCTCC	TTCTCTTTGAGCAGCGGTGT
*GAPDH*	GTCAGTGGTGGACCTGACCT	TGCTGTAGCCAAATTCGTTG
