# Supplementary material for: Mebendazole Mediates Proteasomal Degradation of GLI Transcription Factors in Acute Myeloid Leukemia
Source: Int J Mol Sci. 2021 Oct 1;22(19):10670. doi: 10.3390/ijms221910670 (PMC8508953; doi:10.3390/ijms221910670)
Supplement: Supplementary file 1 [file ijms-22-10670-s001.zip › ijms-1370766-supplementary.pdf]

Supplementary Materials

Supplementary Figures

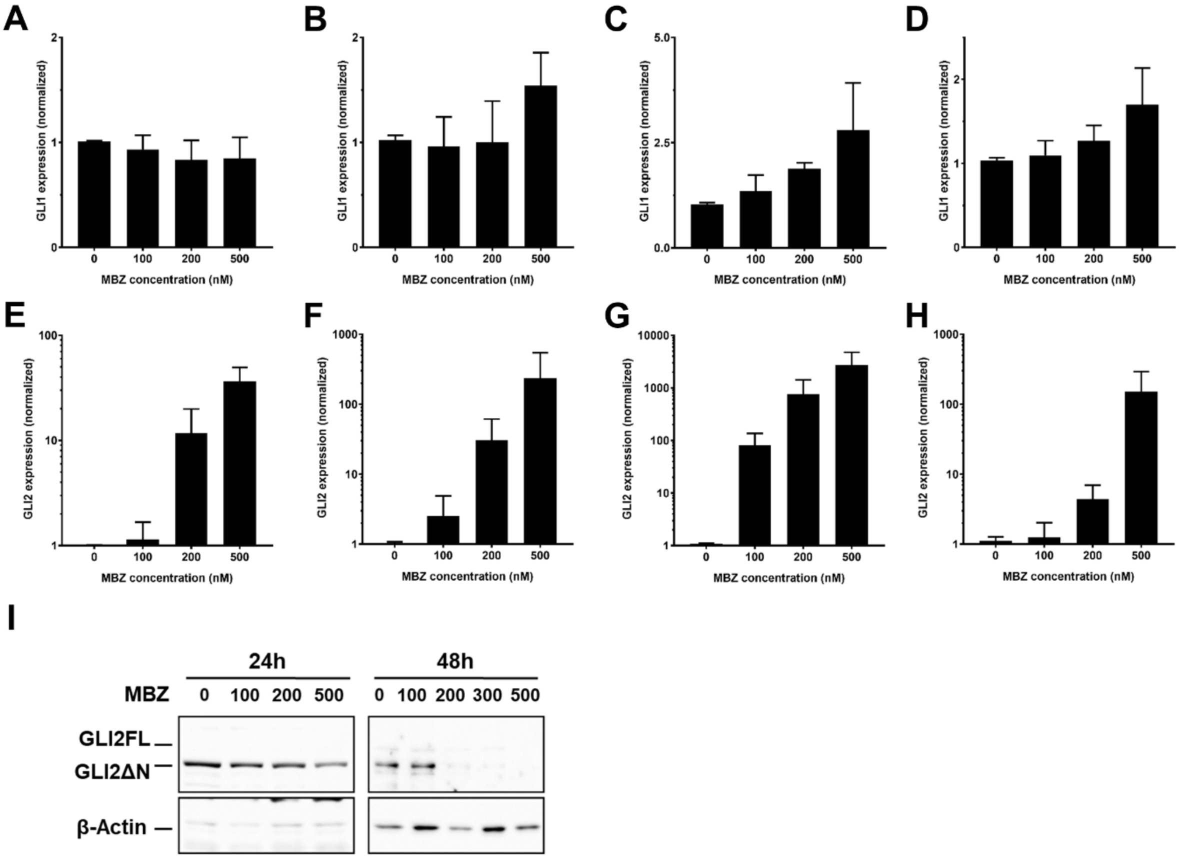

Supplementary Figure S1. Treatment with MBZ does not reduce GLI1 or GLI2 mRNA expression in AML cell lines. For GLI1**,** MV4-11 (**A**), MOLM-13 (**B**), THP-1 (**C**) and OCI-AML3 (**D**) cells were treated with indicated concentrations of Mebendazole (MBZ) for 24h and mRNA expression of GLI1 was determined by qPCR analysis. For GLI2, MV4-11 (**E**), MOLM-13 (**F**), THP-1 (**G**) and OCI-AML3 (**H**) cells were treated with indicated concentrations of Mebendazole (MBZ) for 24h and mRNA expression of GLI2 was determined by qPCR analysis. (**I**) MV4-11 were treated with the indicated concentrations of MBZ or DMSO as solvent control for 24h and 48h, respectively. GLI2 protein expression was examined by western blot analysis. *Error bars represent the mean values ± standard deviation.*

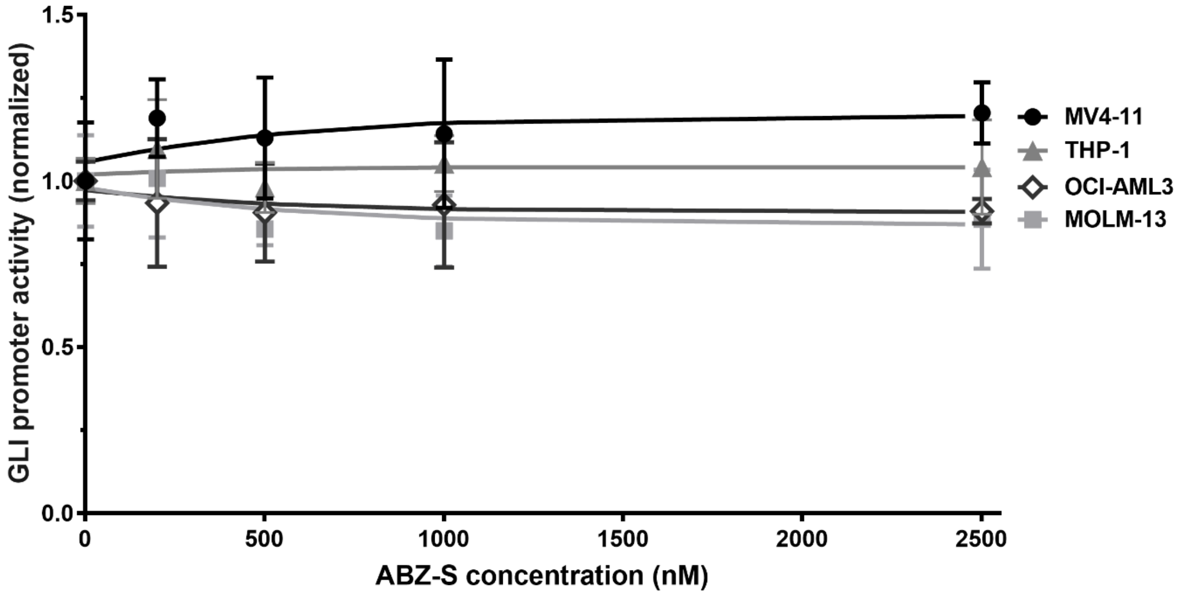

Supplementary Figure S2. Treatment with ABZ does not reduce GLI promoter activity in AML reporter cell lines. The AML reporter cell lines MV4-11, MOLM-13, THP-1 and OCI-AML3 were treated with increasing Albendazole sulfoxide (ABZ-S) concentrations or DMSO as solvent control. The GLI promoter activity was measured after 24h.

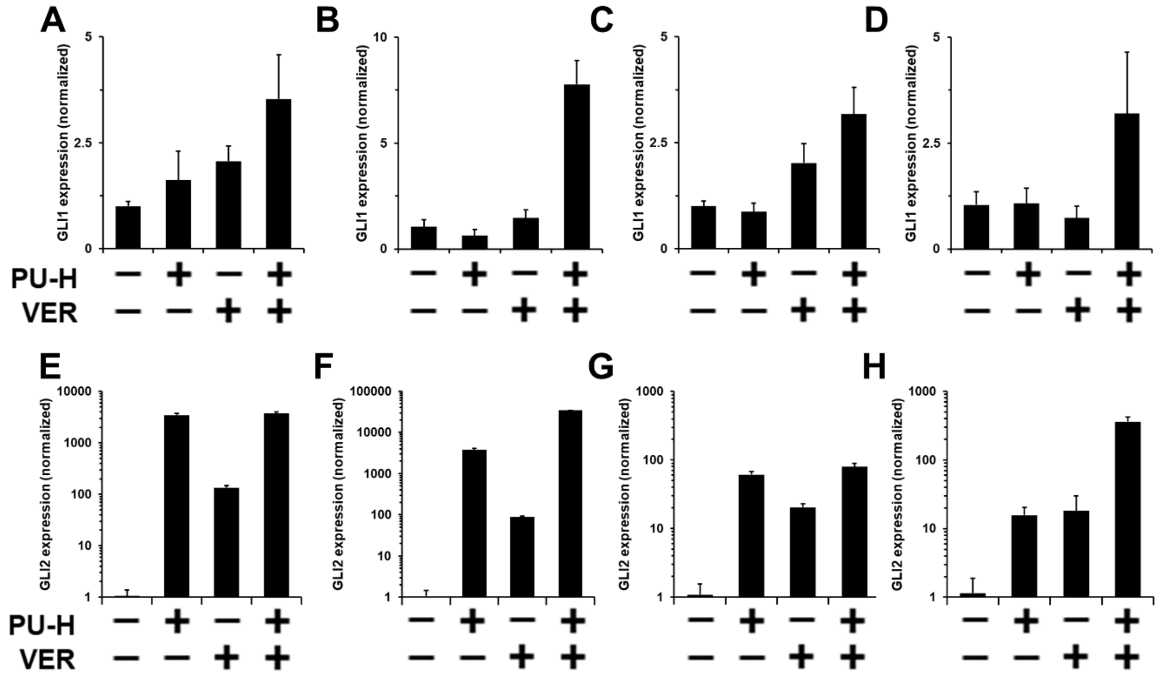

Supplementary Figure S3. Treatment with HSP70 or HSP90 inhibitors does not reduce GLI1 or GLI2 mRNA expression in AML cell lines. For GLI1**,** MV4-11 (**A**), MOLM-13 (**B**), THP-1 (**C**) and OCI-AML3 (**D**) cells were treated with 1 µM PU-H71 (PU-H) and 25 µM VER-155008 (VER) alone and in combination for 24h and mRNA expression of GLI1 was determined by qPCR analysis. For GLI2, MV4-11 (**E**), MOLM-13 (**F**), THP-1 (**G**) and OCI-AML3 (**H**) cells were treated as stated above and mRNA expression of GLI2 was determined by qPCR analysis. *Error bars represent the mean values ± standard deviation.*

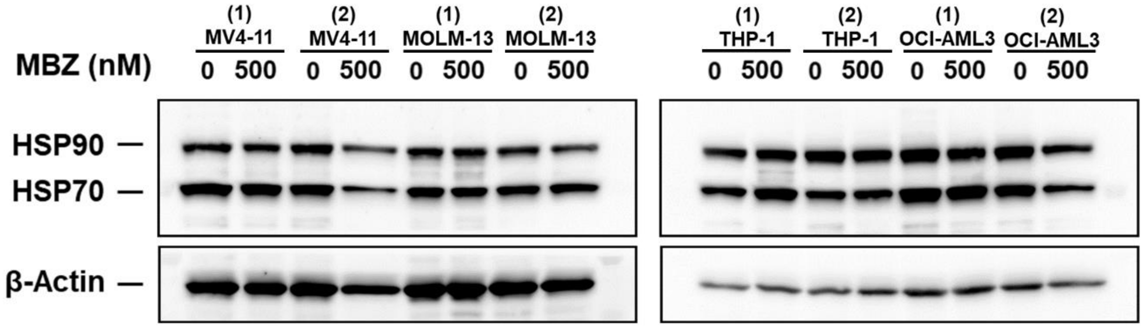

Supplementary Figure S4. Mebendazole treatment does not affect HSP70 or HSP90 protein levels. MV4-11, MOLM-13, THP-1 and OCI-AML3 cells were treated with the indicated concentrations of MBZ alone for 24h. HSP70 and HSP90 protein expression was examined by western blot analysis. β-Actin was used as a loading control.
